# Changes in fetal growth restriction and retinopathy of prematurity during the coronavirus disease 2019 pandemic: A cross-sectional study

**DOI:** 10.1371/journal.pone.0265147

**Published:** 2022-03-16

**Authors:** Shumpei Obata, Riko Matsumoto, Masashi Kakinoki, Shunichiro Tsuji, Takashi Murakami, Takahide Yanagi, Yoshihiro Maruo, Masahito Ohji

**Affiliations:** 1 Department of Ophthalmology, Shiga University of Medical Science, Otsu, Japan; 2 Department of Obstetrics and Gynecology, Shiga University of Medical Science, Otsu, Japan; 3 Department of Pediatrics, Shiga University of Medical Science, Otsu, Japan; University of Abuja/University of Abuja Teaching Hospital, NIGERIA

## Abstract

**Purpose:**

To investigate changes in the number of preterm infants, low birth weight infants, and infants with fetal growth restriction (FGR) or retinopathy of prematurity (ROP) during the coronavirus disease 2019 (COVID-19) pandemic.

**Methods:**

In this retrospective cross-sectional study, we reviewed the medical records of infants born and admitted to the neonatal intensive care unit and growth care unit of Shiga University of Medical Science Hospital before the COVID-19 pandemic (April 1, 2019 to September 30, 2019) and during the pandemic (April 1, 2020 to September 30, 2020). Medical records of infants’ mothers were also collected. Preterm infants, low birth weight infants, infants with FGR, infant and maternal factors associated with FGR, and infants requiring treatment for ROP were compared between the two periods.

**Results:**

There were fewer infants born at < 28 weeks of gestation, infants with birth weight < 1,500 g, and infants with FGR during the pandemic period than the pre-pandemic period (pre-pandemic: n = 4 vs. during pandemic: n = 0, P = 0.048; pre-pandemic: n = 15 vs. during pandemic: n = 6, P = 0.02; and pre-pandemic: n = 31 vs. during pandemic: n = 12, P = 0.0002, respectively). There were no significant differences in any infant or maternal factors associated with FGR. The number of infants requiring treatment for ROP decreased during the pandemic, although this difference was not statistically significant (pre-pandemic: n = 3 vs. during pandemic: n = 0, P = 0.08).

**Conclusions:**

Our findings showed a reduction in the number of infants with FGR during the COVID-19 pandemic. The number of infants born at < 28 weeks of gestation and infants with birth weight < 1,500 g also decreased during the pandemic period. There was a trend toward fewer infants requiring treatment for ROP during the COVID-19 pandemic.

## Introduction

The number of preterm infants and low birth weight infants has reportedly been decreasing during lockdowns or states of emergency owing to the coronavirus disease 2019 (COVID-19) pandemic [1–6]. The underlying mechanisms have been speculated to include potential reduction in work-related stresses, possible alleviation of physical strain related to work, and fewer infections [4]. In Japan, a state of emergency was declared on April 7, 2020, such that people refrained from going out in public to prevent infection; this resulted in a major change in lifestyle. The pandemic and resulting social intervention provided a unique opportunity to evaluate the effects through a natural experiment [7].

In Japan, fetal growth restriction (FGR) is defined as a child with birth weight < –1.5 standard deviation (SD), compared with standard weight [8]. FGR is also defined as a child with birth weight below the 10th percentile, according to the American College of Obstetricians and Gynecologists [9]. FGR has a substantial impact on infant outcomes. Because there is no evidence-based treatment for FGR, it is important to prevent the onset of FGR. Infant factors that can cause FGR include genetic etiologies, structural disorders, and congenital viral infections. Maternal factors that can cause FGR include substance use and abuse (tobacco and alcohol), placental disorders and umbilical cord abnormalities, pregestational diabetes mellitus, renal insufficiency, autoimmune disease, hypertensive disorders of pregnancy, and antiphospholipid antibody syndrome [9]. Lifestyle changes during the COVID-19 pandemic may have reduced the number of infants with FGR. Several reports have shown that the number of preterm infants and low birth weight infants has been decreasing during the pandemic [1–6]. To our knowledge, only one report has investigated the number of infants with FGR, which did not change during the pandemic [6]. Otherwise, there have been no extensive investigations of the changes regarding infant and maternal factors associated with FGR. Notably, FGR is an independent risk factor for retinopathy of prematurity (ROP) [10], and the number of infants requiring treatment for ROP may have changed because of the altered number of infants with FGR. Here, we evaluated changes in the number of infants with FGR, along with potentially influential infant and maternal factors. We also evaluated the number of infants with ROP (with and without a need for treatment) between the periods before and during the COVID-19 pandemic.

## Methods

### Study design and patients

This retrospective cross-sectional study was approved by the Institutional Review Board/Ethics Committee of Shiga University of Medical Science (Otsu, Japan), and an opt-out consent process was used. All procedures performed in this study involving human participants were in accordance with the ethical standards of the institutional and/or national research committee and with the 1964 Declaration of Helsinki and its later amendments or comparable ethical standards. Medical records were collected for infants who were born and admitted to the neonatal intensive care unit (NICU) and growth care unit (GCU) of Shiga University of Medical Science Hospital during the pre-COVID-19 pandemic period (“pre-pandemic”; April 1, 2019 to September 30, 2019) and during the COVID-19 pandemic period (“during pandemic”; April 1, 2020 to September 30, 2020). The medical records of infants’ mothers were also collected. The following infant factors were investigated: sex, gestational age at birth, birth weight (g/SD), and Apgar scores at 1 and 5 min. The following maternal factors were investigated: gestational age, childbirth history, fertility treatment history, mode of delivery, threatened preterm labor, premature rupture of membranes, use of ritodrine hydrochloride, and use of steroid treatment.

### Preterm infants, low birth weight infants, and FGR

Preterm births were categorized as premature birth at < 28 weeks of gestation, < 34 weeks, and < 37 weeks [11]. Birth weight was categorized as low birth weight (< 2,500 g), very low birth weight (< 1,500 g), and extremely low birth weight (< 1,000 g) [11]. In accordance with Japanese guidelines [8], FGR was defined as a child with birth weight < –1.5 SD, compared with standard weight. In accordance with a previous report [9], we investigated possible infant factors associated with FGR (e.g., genetic etiologies, structural disorders, and congenital viral infections) and possible maternal factors associated with FGR (e.g., substance use and abuse [tobacco and alcohol], placental disorders and umbilical cord abnormalities, pregestational diabetes mellitus, renal insufficiency, autoimmune disease, hypertensive disorders of pregnancy, and antiphospholipid antibody syndrome).

### ROP

Medical records were reviewed to determine the number of infants who required ROP screening, the total number of ophthalmologic examinations, the number of ophthalmologic examinations per infant, the number of infants who developed ROP, and the number of infants who required ROP treatment. The screening criteria for ROP at Shiga University of Medical Science Hospital followed the guidelines proposed by the American Academy of Ophthalmology and the Association for Pediatric Ophthalmology and Strabismus, with some modifications [12]. Briefly, these criteria are birth at < 34 weeks of gestation or birth weight < 1800 g. Additionally, infants who were considered to be at high risk of ROP because of receiving high-concentration oxygen therapy were screened, regardless of whether they met the above criteria.

### Statistical analysis

Statistical analyses were performed using GraphPad Prism 8 software (GraphPad Software, Inc., La Jolla, CA, USA). The results are expressed as mean ± SD for continuous variables. Normality was assessed using the Shapiro–Wilk test; each factor was then compared between groups (pre-pandemic and during pandemic) using the Student *t-*test or Mann–Whitney U test. For comparisons of categorical data, Fisher’s exact test was performed. The retrospective power was calculated for the Fisher’s exact test to compare the proportion of infants with FGR between groups because we were unable to estimate the proportion of FGR at our hospital and were unable to calculate a priori sample size. Multiple logistic regression analysis was also conducted to determine whether the probability of FGR decreased during the pandemic when controlling for confounding factors (hypertensive disorders of pregnancy, pregestational diabetes mellitus and renal insufficiency [9]). P-values < 0.05 were considered statistically significant.

## Results

### Baseline characteristic of infants and mothers

Eighty-one infants from 76 mothers were admitted to the NICU or GCU during the pre-pandemic period. Ninety infants from 76 mothers were admitted to the NICU or GCU during the pandemic period. All data are provided in the S1 Dataset. The baseline characteristics of infants and their mothers are shown in Tables 1 and 2. The number of infants from multiple births admitted to the NICU or GCU was similar between the two periods (pre-pandemic: n = 22 vs. during pandemic: n = 25, P > 0.99; Table 3).

**Table 1:** Baseline characteristics of infants in this study.

	Pre-pandemic, 2019	During pandemic, 2020	P-value
	81 infants	90 infants	
Sex, female	46 (57)	49 (54)	0.89
Gestational age, weeks	35.4 ± 3.8	35.9 ± 2.6	0.76
Birth weight, g	2114 ± 727	2312 ± 618	0.046
SD of birth weight, compared with standard weight	-0.84 ± 1.4	-0.34 ± 1.2	0.02
Fetal growth restriction	31 (38)	12 (13)	0.0002
Apgar score at 1 minute	6.9 ± 2.0	7.3 ± 1.8	0.12
Apgar score at 5 minutes	8.7 ± 0.9	8.6 ± 1.1	0.79
Preterm birth < 37 weeks	47 (58)	58 (64)	0.53
Preterm birth < 34 weeks	22 (27)	22 (24)	0.73
Preterm birth < 28 weeks	4 (5)	0 (0)	0.048
Birth weight < 2500 g	55 (68)	59 (66)	0.87
Birth weight < 1500 g	15 (19)	6 (7)	0.02
Birth weight < 1000 g	6 (7)	2 (2)	0.15

Data are shown as number (%) or mean ± standard deviation (SD).

**Table 2:** Baseline characteristics of mothers in this study.

	Pre-pandemic, 2019	During pandemic, 2020	P-value
	76 mothers	76 mothers	
Gestational age, years	33.5 ± 5.1	33.5 ± 5.7	0.88
Gravidity	2.0 ± 1.1	2.3 ± 1.7	0.98
Parity	0.72 ± 0.76	0.79 ± 1.0	0.85
Assisted reproductive technology	21 (28)	28 (37)	0.30
Cesarean section	40 (53)	48 (63)	0.25
Threatened preterm labor	21 (28)	20 (26)	> 0.99
Premature rupture of membrane	18 (24)	15 (20)	0.69
Use of ritodrine hydrochloride	23 (30)	25 (33)	0.86
Use of steroid treatment	16 (21)	22 (29)	0.35

Data are shown as number (%) or mean ± standard deviation.

**Table 3:** Infant factors associated with fetal growth restriction.

	Pre-pandemic, 2019	During pandemic, 2020	P-value
	31 infants	12 infants	
Multiple gestation	6 (19)	1 (8)	0.65
Genetic disorders and/or structural disorders	8 (26)	2 (17)	0.70
Congenital viral infections	0 (0)	2 (17)	0.07

Data are shown as number (%).

### Preterm infants and low birth weight infants

Infant birth weight was greater during the pandemic period than during the pre-pandemic period (2312 g vs. 2114 g, P = 0.046). The number of infants born at < 28 weeks’ gestation decreased significantly from 4 during the pre-pandemic period to 0 during the pandemic period (P = 0.048). The number of very low birth weight infants (< 1,500 g) decreased significantly from 15 during the pre-pandemic period to 6 during the pandemic period (P = 0.02). The number of infants with FGR decreased significantly from 31 during the pre-pandemic period to 12 during the pandemic period (P = 0.0002; the retrospective power for this test was 95.2%).

### Infant and maternal factors associated with FGR

Infant factors and maternal factors that may be associated with FGR are shown in Tables 3 and 4, respectively. There were no statistically significant differences between the pre-pandemic and pandemic periods in any infant or maternal factors. Multivariate logistic regression analysis showed that only the period during the pandemic was significantly associated with a decreased number of infants with FGR (P = 0.0005, Table 5).

**Table 4:** Maternal factors associated with fetal growth restriction.

	Pre-pandemic, 2019	During pandemic, 2020	P-value
	30 mothers	11 mothers	
Smoking (+/–/unknown)	7/18/5	3/7/1	0.83
Alcohol (+/–/unknown)	0/25/5	0/10/1	-
Placental disorders and umbilical cord abnormalities	0 (0)	0 (0)	-
Pregestational diabetes mellitus	2 (7)	1 (9)	0.79
Renal insufficiency	1 (3)	0 (0)	0.54
Autoimmune disease	0 (0)	0 (0)	-
Hypertensive disorders of pregnancy	2 (7)	0 (0)	0.38
Antiphospholipid antibody syndrome	0	0	-
Disease of the thyroid	4 (13)	0 (0)	0.20

Data are shown as number or number (%).

The symbol “-” in the table indicates Fisher’s exact test could not be performed.

**Table 5:** Logistic regression analysis of risk factors for infants with FGR.

Explanatory variables	Odds ratio	95% CI	P Value
During pandemic	0.25	0.11–0.54	0.0005
No: 0, Yes: 1			
Hypertensive disorders of pregnancy	3.6	0.54–30	0.19
No: 0, Yes: 1			
Pregestational diabetes mellitus	0.66	0.10–2.7	0.61
No: 0, Yes: 1			
Renal insufficiency	0.77	0.07–7.1	0.82
No: 0, Yes: 1			

CI, confidence interval.

### ROP

**Fig 1:**
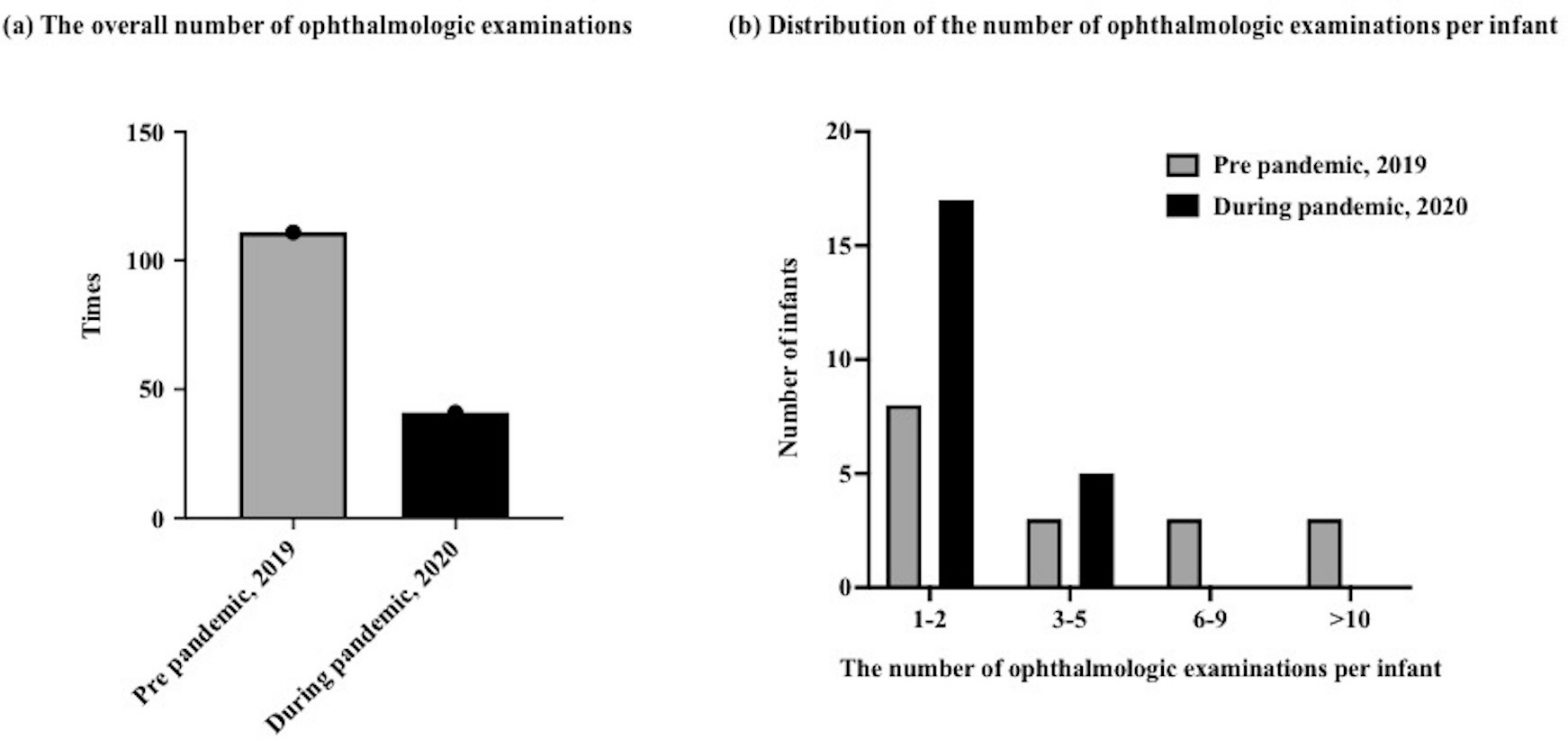
Number of ophthalmologic examinations. (a) Overall number of ophthalmologic examinations. (b) Distribution of the number of ophthalmologic examinations per infant.

**Fig 2:**
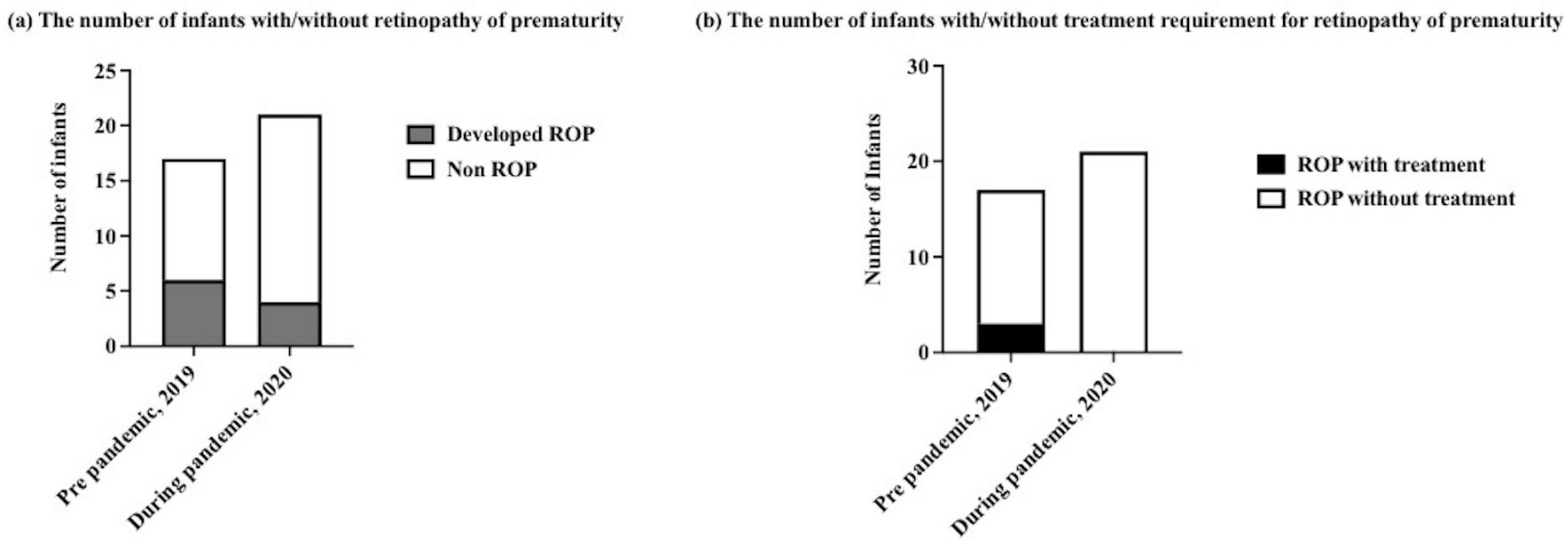
Retinopathy of prematurity. (a) Number of infants with/without retinopathy of prematurity. (b) Number of infants with/without retinopathy of prematurity requiring treatment.

ROP screening was required in 17 infants (21%) during the pre-pandemic period and in 21 infants (23%) during the pandemic period. The overall number of ophthalmologic examinations decreased from 111 to 41, and there were fewer than five ophthalmologic examinations per infant among all infants during the pandemic period (Fig 1). The number of infants who developed ROP decreased slightly from 6 to 4 (P = 0.29; Fig 2A). The number of infants requiring ROP treatment decreased from 3 to 0, although this difference was not statistically significant (P = 0.08; Fig 2B).

## Discussion

The number of infants born at < 28 weeks and the number of very low birth weight (< 1,500 g) infants both decreased in the current study, consistent with the findings of previous studies [2–4, 6]. Furthermore, the number of infants with FGR decreased during the pandemic period, as compared with the pre-pandemic period; this contrasted with the findings of Matheson et al [6]. We observed a trend toward fewer infants requiring ROP treatment during the pandemic, presumably because of the decreased number of preterm infants, low birth weight infants, and infants with FGR.

### Decreased number of preterm infants and low birth weight infants during the COVID-19 pandemic

The number of preterm infants has reportedly decreased during the COVID-19 pandemic [1–3, 5, 6]. We found a similar decrease in the number of infants born at < 28 weeks, consistent with the findings of previous reports [2, 3, 5, 6]. However, we found no differences in the rates of threatened preterm labor or use of ritodrine hydrochloride (a drug used during threatened preterm labor). In past reports, factors underlying the lower number of preterm infants were presumably changes in lifestyle (e.g., reduced risk of infections such as influenza, less physically demanding work, less shift work, less work-related stress, improved sleep duration, maternal engagement in outdoor and indoor exercise, and increased social support) because of business closures and reduced air pollution [1, 3]. The association between these factors and the decrease in preterm births is unclear, however, because these factors were not assessed in this study.

We found that the number of infants with birth weight < 1,500 g decreased significantly during the pandemic period, as compared with the pre-pandemic period. The number of infants with birth weight < 1,000 g decreased, although this difference was not statistically significant (presumably because of the small number of such infants in both periods). However, the number of infants with birth weight < 2,500 g was unaffected by the pandemic. Previous reports have shown decreases in the number of infants with birth weights < 1,000 g and < 1,500 g [4] whereas no reports have described the number of infants with birth weight < 2,500 g. Overall, our findings and the published literature suggest that there are likely to be fewer infants with birth weight < 1,500 g during the COVID-19 pandemic.

### Reduced FGR incidence

In the current study, the number of infants with FGR decreased significantly from 31 (38%) during the pre-pandemic period to 12 (13%) during the pandemic period. Various causes of FGR have been reported [9]. We evaluated many factors in this study; notably, we found no significant differences in any infant or maternal factors that potentially contribute to FGR. The reduced number of infants with FGR may be related to factors other than those examined in this study, such as a decrease in subclinical infections because of infection control measures. Asymptomatic maternal infections can cause intrauterine infections via vertical transmission, initiating a cascade that leads to preterm birth [13]. The COVID-19 pandemic might have changed health behaviors, resulting in an unexpected reduction in seasonal influenza transmission [14]. Isolation owing to lockdowns, physical distancing, and enhancement of hygiene awareness (e.g., hand-washing and mask-wearing) can serve to reduce contact with pathogens, thus reducing the risk of infection. The reduced incidence of FGR observed in this study suggests that hygiene measures and anticipatory behavioral changes might have contributed to improved pregnancy outcomes.

A report from Australia using total birth data from three obstetric clinics showed no change in the number of infants with FGR between the pre-pandemic and pandemic periods [6]. A report from Australia [6] compared the period from January to September before the pandemic (2019) and during the pandemic (2020), which was similar in design to our comparison study. In the report from Australia, the proportion of preterm infants and infants with FGR was compared using total births as the denominator. Our study design differed in that we compared the proportion of infants with FGR using infants admitted to NICU as the denominator. Because our study focused on infants with severe conditions who would be admitted to the NICU, it may have been possible to detect changes in FGR. The other discrepancy with our results may be related to differences in ethnicity and COVID-19 infection control measures among countries. There may also be an influence as a result of the distinct definitions of FGR (i.e., Japanese guidelines in Japan [8] and American College of Obstetricians and Gynecologists guidelines in Australia [9]).

### Reduced incidence of ROP requiring treatment

The requirement for ROP treatment may have been reduced during the COVID-19 pandemic, although no other reports have shown similar results. Preterm birth, low birth weight, and FGR are reported risk factors for ROP [10, 15–18]. The number of infants born at < 28 weeks, low birth weight infants (especially those with birth weight < 1500 g), and infants with FGR all decreased, which may have led to the trend toward reduced incidence of ROP requiring treatment.

### Limitations

First, this was a retrospective study with a small sample size. Second, the current study included infants admitted to the NICU and GCU at a university hospital, as well as their mothers; this population differs from the populations in studies that have used a hospital’s entire birth database [1–5]. However, because most patients with severe conditions (e.g., very premature infants and very low birth weight infants) are admitted to the NICU or GCU, these infants were not excluded in the present study. Finally, the retrospective nature of this study made it difficult to examine factors not included in the medical records, such as the levels of rest and stress as well as asymptomatic infections in pregnant women, as well as environmental factors (e.g., air pollution).

## Conclusions

This study revealed decreases in the number of infants born at < 28 weeks, infants with birth weight < 1500 g, and infants with FGR during the COVID-19 pandemic period. However, the specific cause of these changes remains unclear. Further research is warranted to elucidate why the number of infants with FGR decreased during the pandemic.

## Supporting information

S1 Dataset Data included in the analysis. (XLSX)
